# Supplementary figures and images for: Soft Sweeps Are the Dominant Mode of Adaptation in the Human Genome
Source: Mol Biol Evol. 2017 May 8;34(8):1863–77. doi: 10.1093/molbev/msx154 (PMC5850737; doi:10.1093/molbev/msx154)

Figure S1

CEU

A

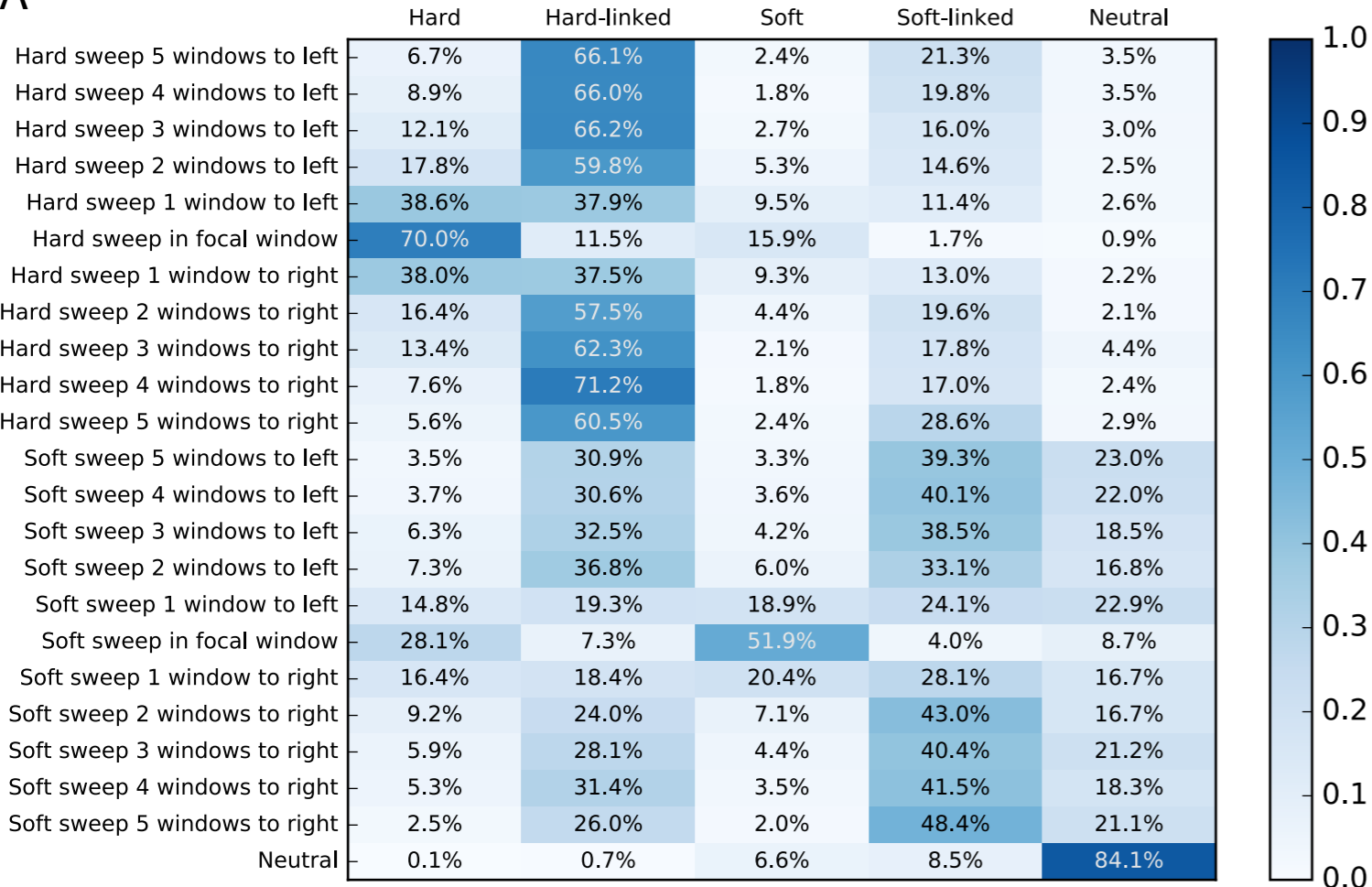

B

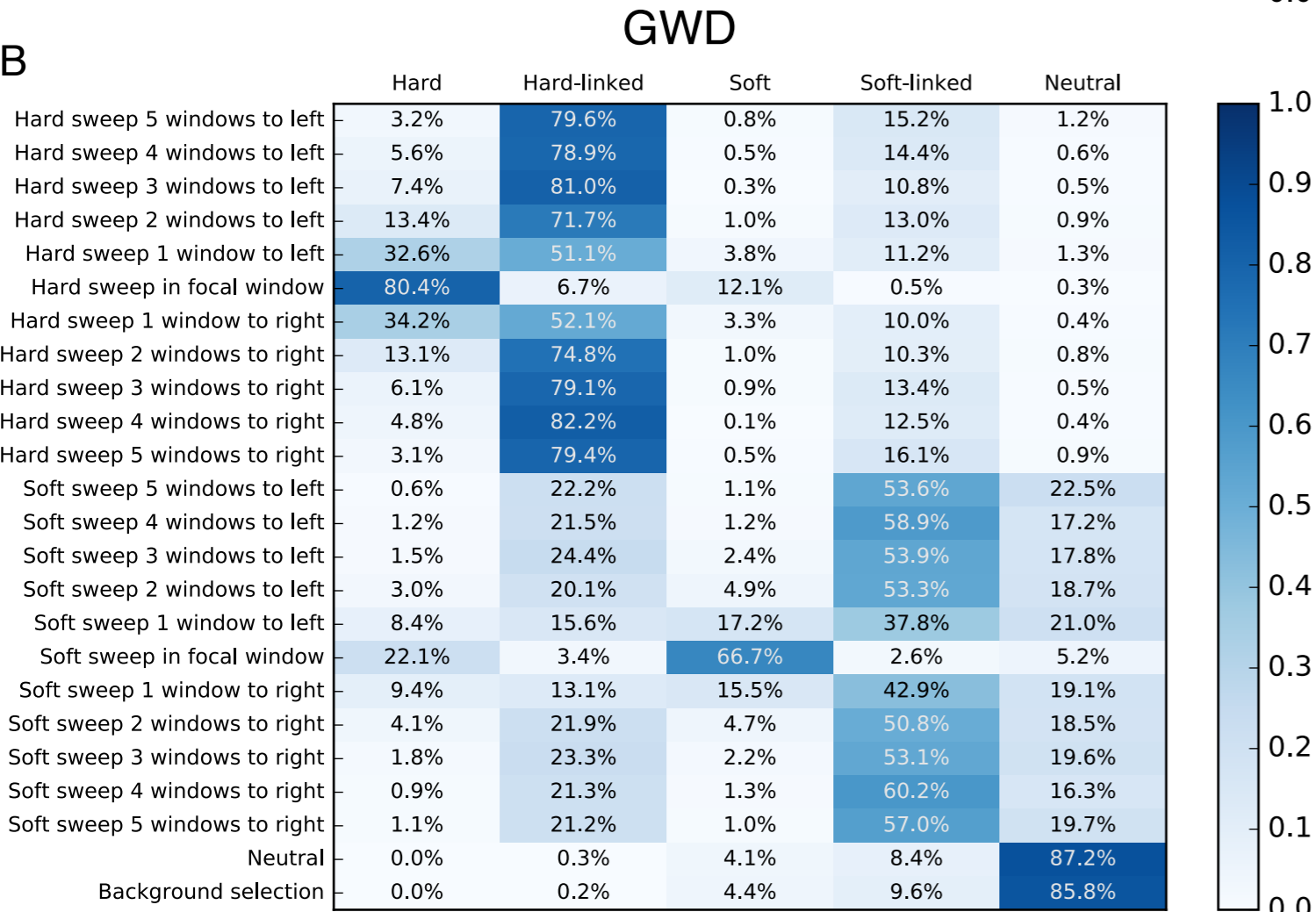

C

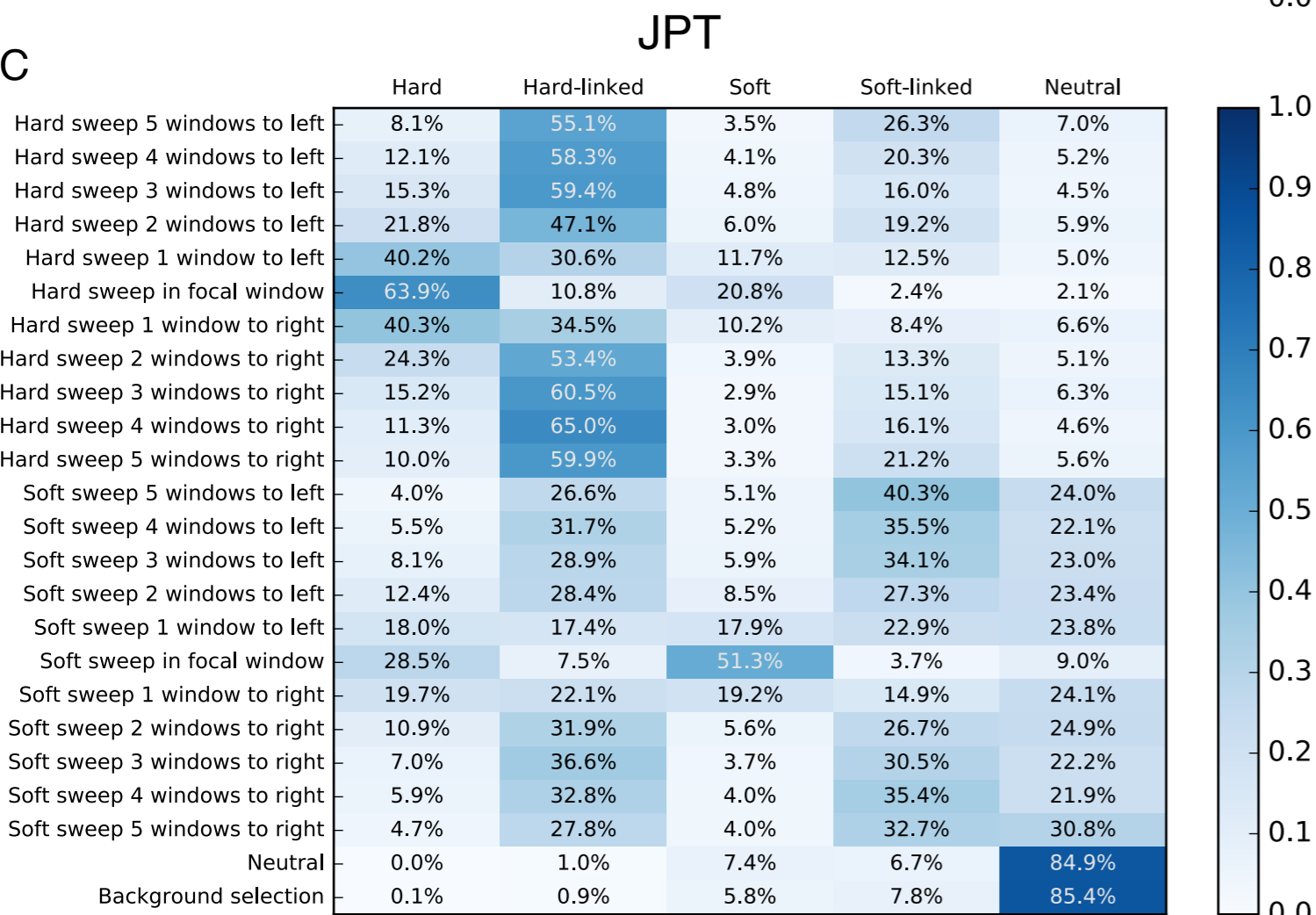

LWK

D

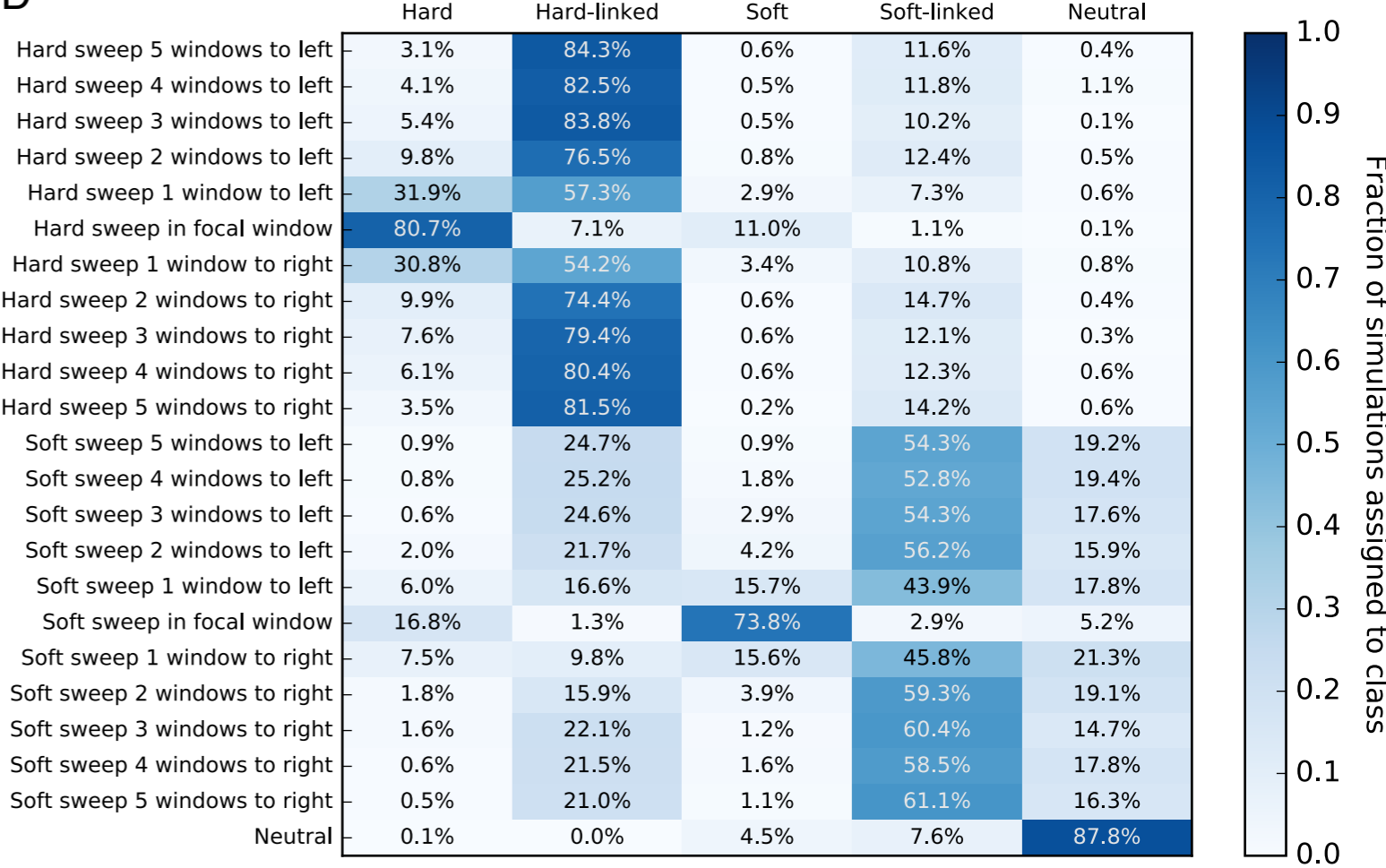

E

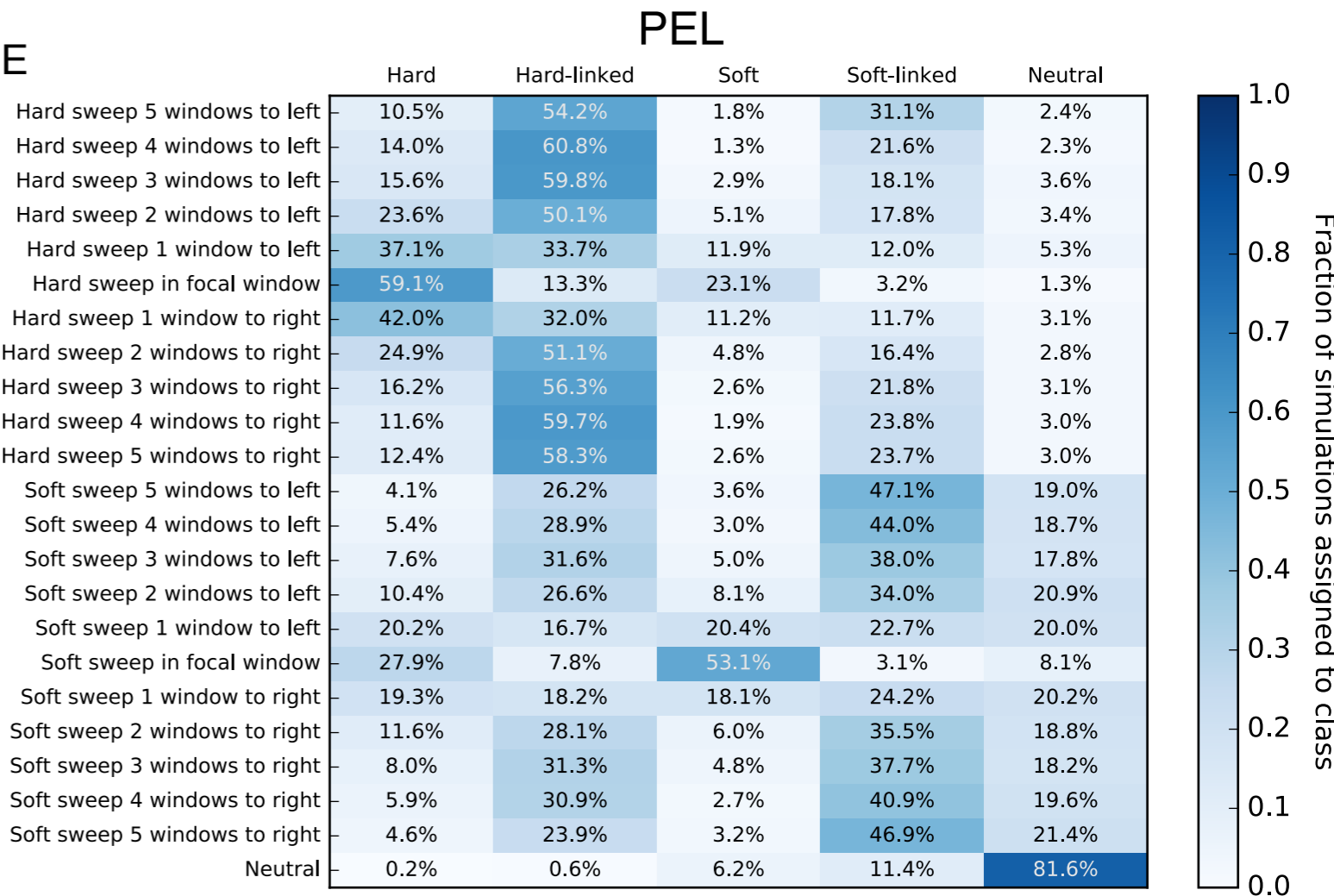

F

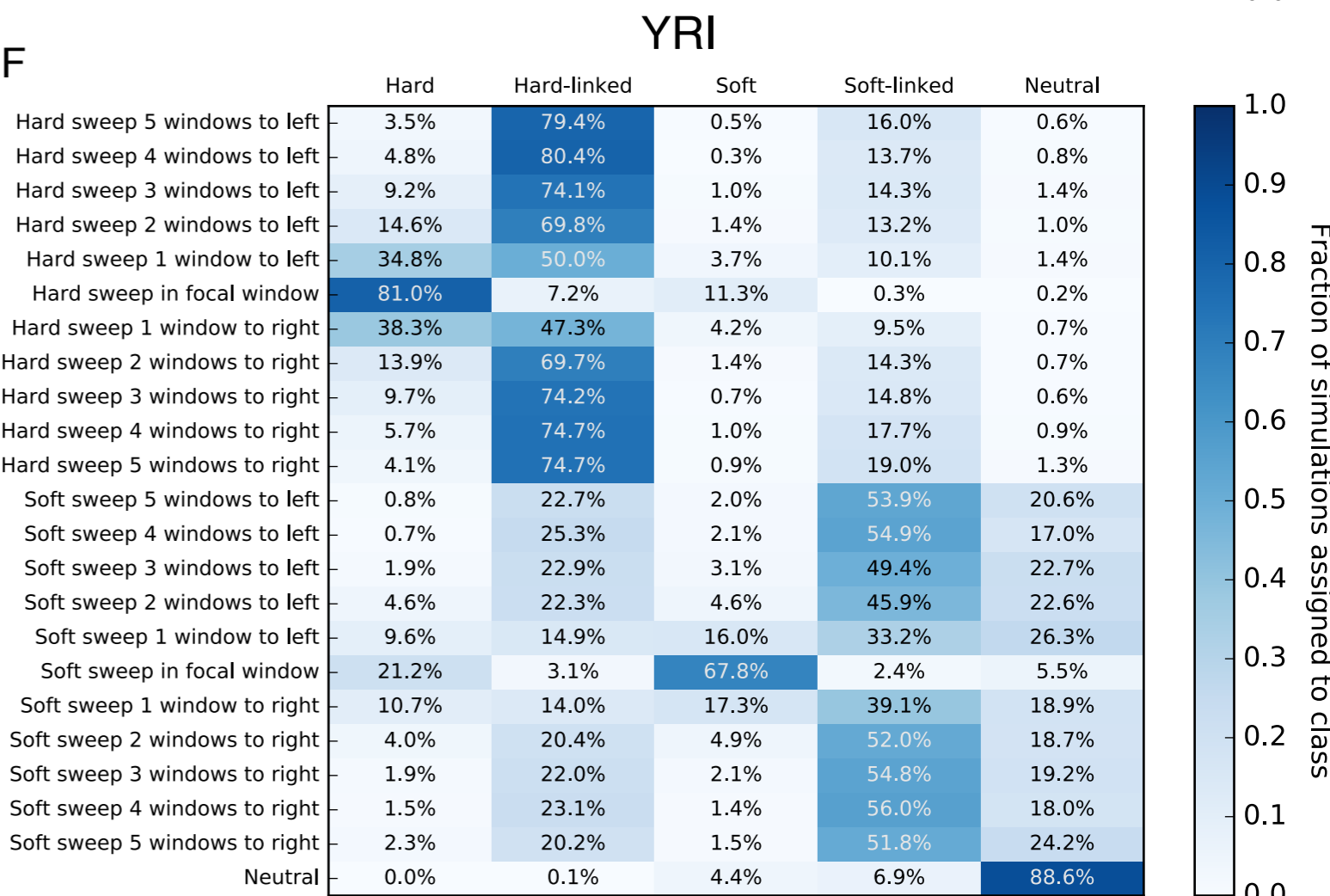

Supplement: Supplementary Data [file msx154_Supp.zip › supplementary_fig_S1.pdf]

Figure S2

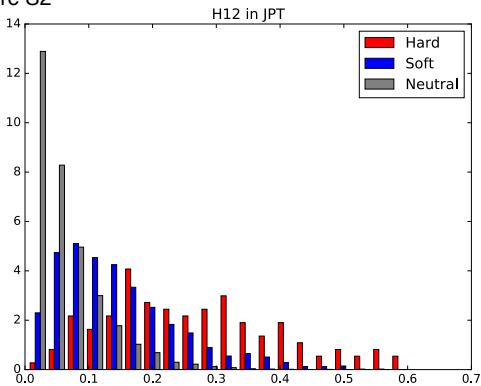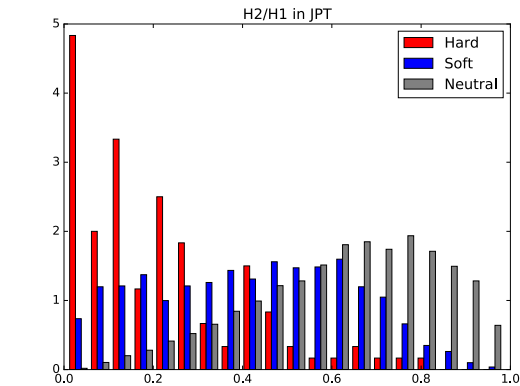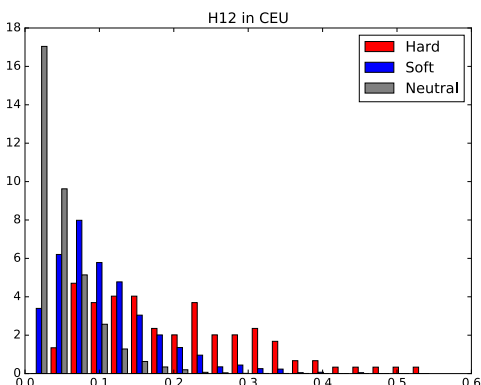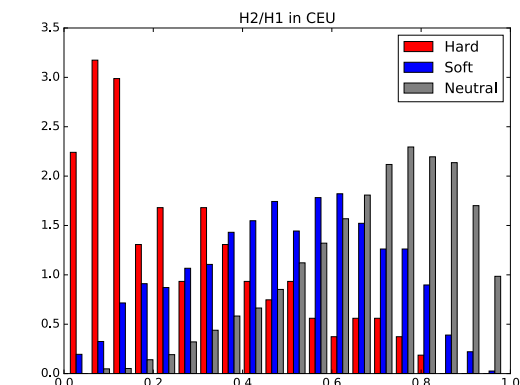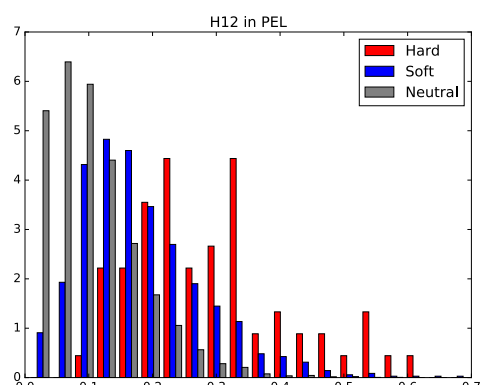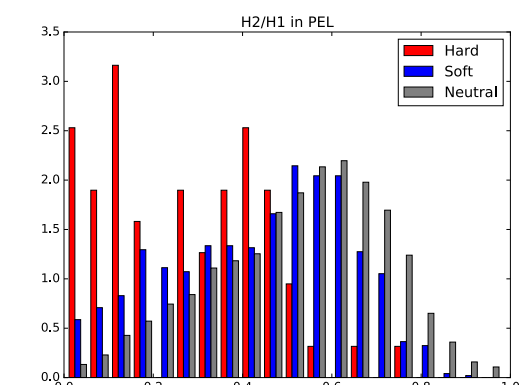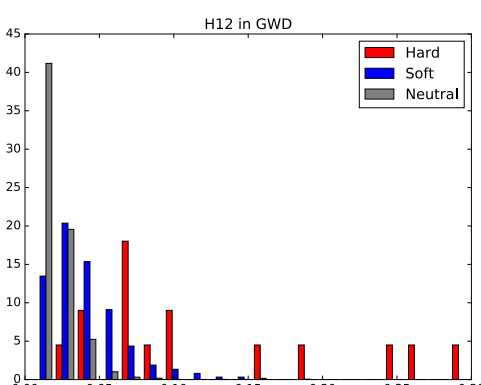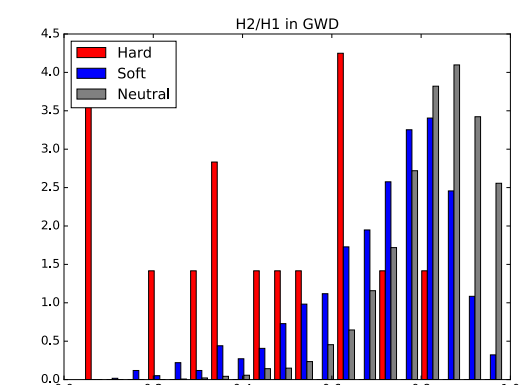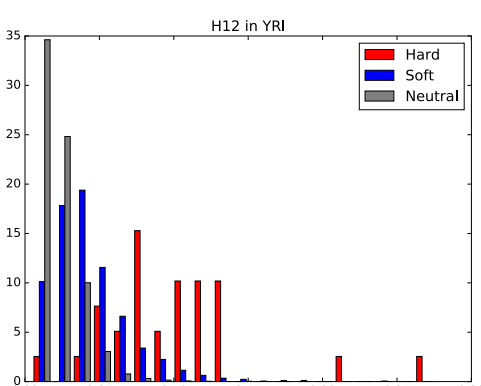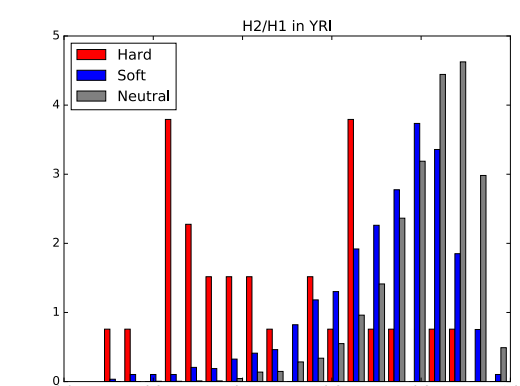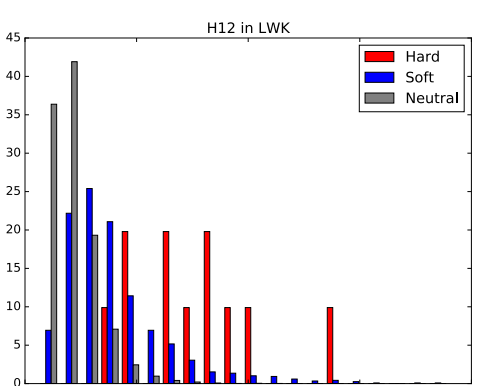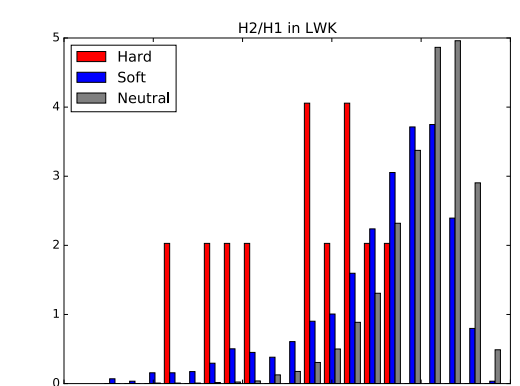

Supplement: Supplementary Data [file msx154_Supp.zip › supplementary_fig_S2.pdf]

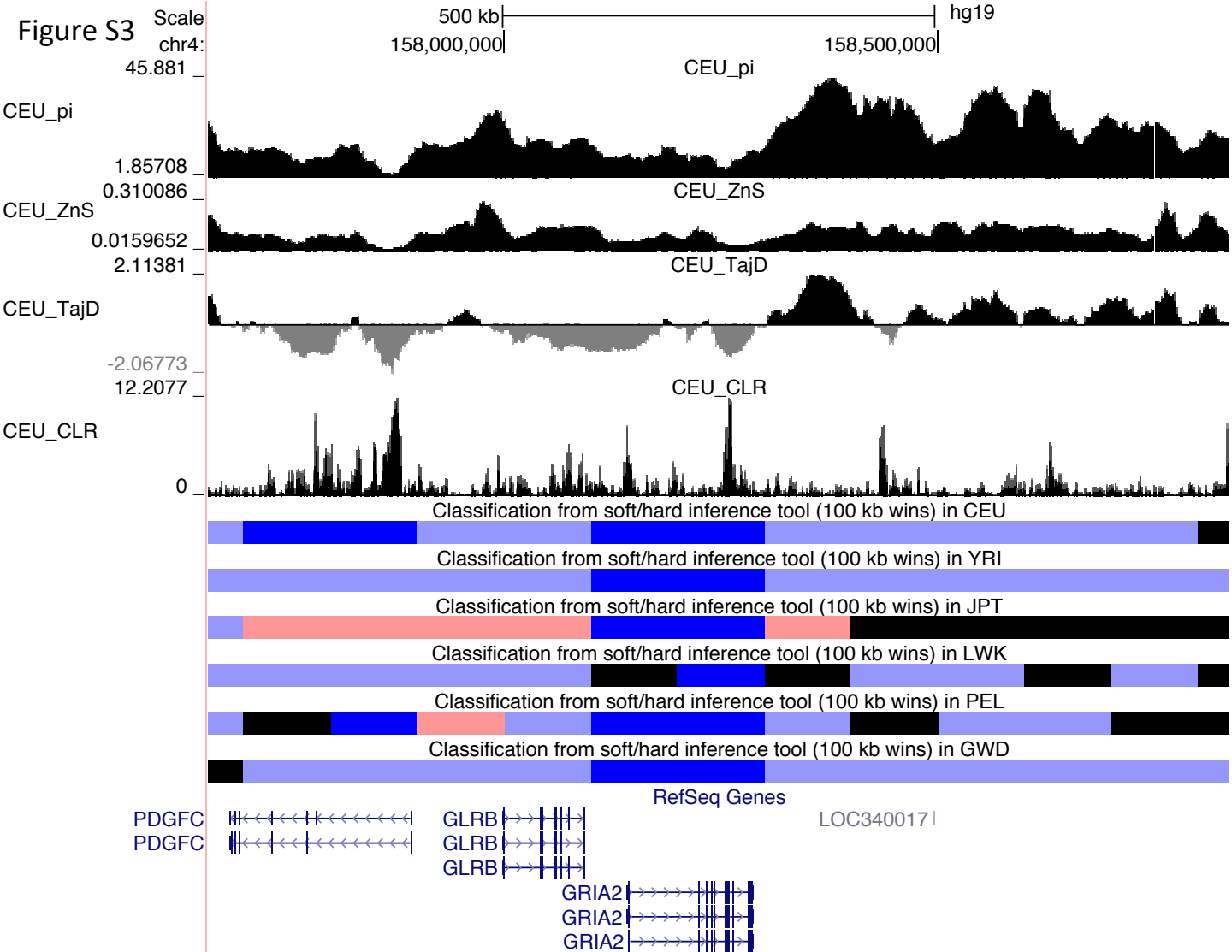

Supplement: Supplementary Data [file msx154_Supp.zip › supplementary_fig_S3.pdf]

Figure S4

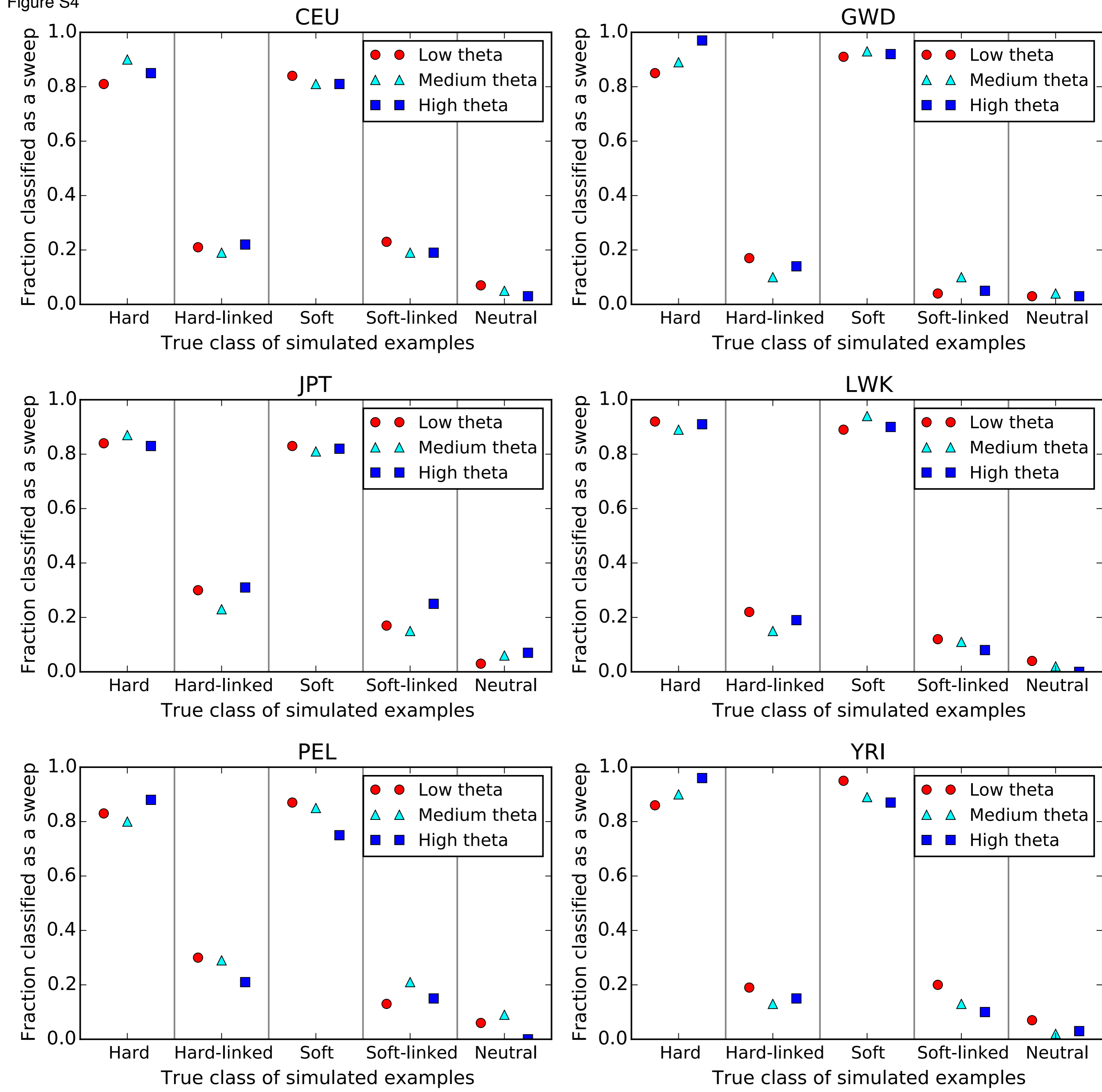

Supplement: Supplementary Data [file msx154_Supp.zip › supplementary_fig_S4.pdf]
